# Adopting Data to Care to Identify and Address Gaps in Services for Children and Adolescents Living With HIV in Mozambique

**DOI:** 10.9745/GHSP-D-23-00130

**Published:** 2024-04-29

**Authors:** Belmiro Sousa, Sergio Chiale, Hayley Bryant, Lisa Dulli, Tanya Medrano

**Affiliations:** a FHI 360, Maputo, Mozambique.; b CARE International, Maputo, Mozambique.; c FHI 360, Durham, NC, USA.

## Abstract

**Background:**

The Data to Care (D2C) strategy uses multiple sources of complementary data on HIV clients and related services to identify individuals with gaps in HIV treatment. Although D2C has been widely used in the United States, there is no evidence on its use in other settings, such as countries most affected by the epidemic.

**Strategy Implementation:**

The D2C strategy was implemented within the context of a project that provided community-based support to children and adolescents living with HIV (C/ALHIV) in Mozambique. A data tracking tool and a standard operating procedure manual for local partner community organizations and health care facilities were developed to support the effort. Project staff met with local project implementing partners to discuss and coordinate the intervention in pilot health facilities.

**Strategy Piloting:**

The project initiated a pilot D2C intervention in 2019, working with 14 health facilities across 5 additional districts within 1 province. COVida project data were compared with clinical data from facilities serving C/ALHIV. The D2C intervention identified gaps in HIV treatment for a substantial number of C/ALHIV, and targeted support services were provided to address those gaps. Viral load (VL) monitoring was added in March 2020. Before the intervention, 71% of C/ALHIV reported to be on HIV treatment by their caregivers were documented as on treatment in health facilities. Support interventions targeted those not on treatment, and this proportion increased to 96% within 1 year of implementation. Additionally, 12 months later, the proportion of C/ALHIV with a documented VL test increased from 52% to 72%.

**Conclusion:**

Introducing the D2C pilot intervention was associated with substantial improvements in HIV treatment for C/ALHIV, including increased linkage to and continuity in treatment and increased VL testing. D2C may be a useful approach to improve health outcomes for C/ALHIV in settings outside of the United States.

## Abstract (precis)

Real-time learning and implementation of the Data to Care strategy within the context of a large HIV intervention program in Mozambique provided a useful opportunity to evaluate, refine, and scale up this evidence-based approach to improve outcomes for children and adolescents living with HIV.

## Teaser

**Key Findings:**

- Children and adolescents living with HIV (C/ALHIV) in Mozambique were being served by both facility-based health services and community-based supportive services, but there was little collaboration between them.
- A joint data review between the projects supporting the 2 services identified discrepancies between their records, with missing or incorrect data on indicators, such as numbers of C/ALHIV on treatment and viral load (VL) testing status.
- A Data to Care (D2C) intervention was piloted in which staff from both facility-based and community-based projects conducted routine joint data reviews to correct and complete their data to improve care for C/ALHIV and guide actions for health care providers to reengage C/ALHIV or provide additional services as needed.
- By the end of the pilot intervention, HIV treatment retention and VL testing increased, and the percent agreement between community and health facility data improved. However, the proportion of C/ALHIV in treatment with an undetectable VL decreased.

**Key Implication:**

- The D2C strategy has the potential to be adopted in settings outside the United States, adding to the set of tools available to improve HIV treatment outcomes in countries most affected by the HIV epidemic.

## BACKGROUND

Despite significant gains in HIV care and treatment, far too many people living with HIV (PLHIV), particularly children and adolescents (C/ALHIV), do not fully benefit from available services.^1^ Among PLHIV, treatment with antiviral medications to suppress the virus is critical to better health and longevity and permits PLHIV to lead otherwise normal lives. However, C/ALHIV are more likely to discontinue treatment, have poorer adherence to treatment, are less likely to be virally suppressed (indicating treatment success), and are even less likely to have a current viral load (VL) test to monitor treatment compared to adult PLHIV.^2^^,^^3^

Efforts to reach C/ALHIV and meet their particular needs have lagged behind those targeting adults.^4^^,^^5^ In many sub-Saharan countries most affected by the epidemic, programs focusing on C/ALHIV, often in the form of programs for orphans and vulnerable children (OVC), are designed to directly address many of the challenges to optimal HIV treatment.^6^ These projects typically implement multifaceted intervention approaches targeting many of the structural barriers that impede HIV care and treatment.^3^^,^^7^

Despite these efforts, there remain challenges to getting C/ALHIV on HIV treatment, keeping them on treatment, and keeping them virally suppressed. Many of the same social and structural barriers to HIV care and treatment faced by adults living with HIV also impact children and adolescents. In addition, their dependence on others, such as a parent or caregiver, to access health services and take the medications can facilitate their care but can also exacerbate some of these barriers.^3^^,^^8^ Additionally, low-quality programmatic and clinical data make active monitoring and follow-up of C/ALHIV enrolled in treatment services difficult. Inaccurate and incomplete data cause C/ALHIV to be miscategorized as on or off treatment, and missing data on VL tests can hinder appropriate treatment.^9^^–^^11^

Data quality issues and their impact on health care quality in HIV programs are a long-standing, widespread problem. In low- and middle-income countries, strains on health care infrastructures, such as staff shortages, lack of electronic medical record systems that are connected across facilities, reliance on paper-based health record systems, and lack of decentralized laboratory testing facilities, lead to incomplete or delayed recordkeeping, delays in lab results, and data entry errors.^12^ Additionally, patient-level factors rooted in HIV-related stigma can lead C/ALHIV or their caregivers to provide false names and contact information, move from facilities without telling providers, and even withhold relevant information from providers for fear of disclosure.^13^ Missing or inaccurate data can lead to treatment interruption for C/ALHIV, lack of continuity of care, and missed opportunities to intervene early to prevent the development of resistance to first-line treatment regimens.^2^

Missing or inaccurate data can lead to treatment interruption for C/ALHIV, lack of continuity of care, and missed opportunities to intervene early to prevent the development of resistance to first-line treatment regimens.

In global HIV programs, much of the focus on data quality to date has been on the use of data to measure and monitor program progress and performance, such as data for decision-making efforts at program and national levels^10^^,^^14^^–^^24^ or for epidemiological surveillance and research activities.^18^^,^^20^^,^^25^^,^^26^ Though the 2 goals are inextricable, more limited efforts have focused specifically on improving the quality of clinical record data for the purposes of clinical care.^27^

To improve HIV patient treatment and care, the Data to Care (D2C) strategy uses multiple sources of HIV patient data, such as health management information/surveillance data, patient charts, laboratory data, and pharmacy data, to identify PLHIV who have not initiated treatment or have discontinued treatment, to identify treatment gaps, and to link PLHIV to appropriate HIV care services.^28^ Developed by the U.S. Centers for Disease Control and Prevention, the D2C strategy has been demonstrated in the United States to be effective at identifying PLHIV who are not in care, re-engaging them in care, identifying treatment adherence issues, and in some cases, has been shown to improve viral suppression among PLHIV.^29^^–^^37^ Although the D2C strategy has been shown to be an effective strategy to improve retention in the HIV continuum of care in the United States, it has not been widely adopted in other countries.

## ADOPTING THE DATA TO CARE STRATEGY IN MOZAMBIQUE

In Mozambique, the COVida–Juntos Pelas Crianças (meaning Together for Children) project aimed to improve the health, nutritional status, and well-being of OVC, including C/ALHIV, through community-based support services. The COVida project worked with local community-based organizations to train and manage community case workers who provided direct support services to C/ALHIV and their families. These services included counseling, support, and referral to health services, as well as actively linking families with local resources, such as schools and other governmental programs, that provided complementary services to community members. Funded by the U.S. Agency for International Development/U.S. President’s Emergency Plan for AIDS Relief, COVida collaborated directly with another U.S. Agency for International Development-funded project, ESCALA, which provided technical support for pediatric HIV clinical care and treatment services. The COVida project was implemented from 2016 to 2022 in 30 districts across 7 provinces and served 22,032 C/ALHIV in total.

During COVida project implementation, COVida staff identified numerous data issues that hindered their ability to adequately support the C/ALHIV enrolled in their project services. Staff observed that there were considerable discrepancies between health facility data and COVida project data in the numbers of C/ALHIV who were currently on antiretroviral therapy (ART), as well as gaps in data on treatment adherence and VL testing. In response to these data challenges, the COVida project adopted the D2C strategy in Mozambique to ensure appropriate HIV care and treatment for their project participants and ensure all eligible C/ALHIV enrolled in ART had access to the services provided by the COVida project.

The D2C intervention in Mozambique aimed to complement clinical data with COVida programmatic data to identify C/ALHIV who were not on treatment (had discontinued treatment or had started treatment) or who needed critical clinical services, such as VL testing. Additionally, the intervention was designed to identify C/ALHIV enrolled in ART services who would benefit from the support services provided by the COVida project but who had not enrolled in the project.

The D2C intervention was jointly implemented by the COVida project and ESCALA projects. A data tracking tool was developed to support the effort (Supplement). COVida staff met with ESCALA staff and its local implementing partners to discuss and coordinate how to conduct the intervention in specific health facilities in the selected districts. The project also developed a standard operating procedure manual for their local partner community organizations and health care facilities. All documents that contained individually identifying information on patients or project participants were managed according to shared confidentiality agreements between the project and the health facilities.

## PILOTING THE DATA TO CARE INTERVENTION

COVida partners generated lists of C/ALHIV enrolled in the project, and health facility staff checked these data with their electronic patient tracking system. These lists were used to identify children who needed to be followed up by community case managers. Children younger than 18 years were included in these lists only if their caregivers had provided consent to the project or health facility to be contacted in case of treatment interruption. Adolescents ages 18 to 19 years were included in the list if they had provided their own consent to be contacted.

Discrepancies regarding treatment status were identified, and clinic staff and COVida partners worked to update and align information in their 2 respective data systems and identify C/ALHIV in need of intervention. The data that were routinely reviewed included ART status and regimen, VL testing status, and COVida project enrollment status. Depending on the results of the monthly reviews, case management supervisors engaged by COVida could refer C/ALHIV who:

- Had discontinued treatment and were still active participants in COVida to the health facility so they could reinitiate treatment
- Had detectable VL to enhanced adherence counseling at the health facility and have case managers reinforce adherence counseling during home visits
- Were not on an optimized treatment regimen to the health facility to transition to an optimized regimen
- Were eligible/due for a VL test to VL testing

This D2C intervention was piloted in 14 health care facilities located in 5 districts (Zavala, Maxixe, Morrumbene, Massinga, and Vilankulo districts) in Mozambique covering approximately 1,700 C/ALHIV at the time of the pilot. The pilot intervention was implemented from October 2019 to September 2020, though data on VL monitoring were not added to the efforts until March 2020. Data on treatment status, VL testing status and results, and enrollment in the COVida project were monitored over the course of the project.

### Enrolled on ART

**FIGURE 1:**
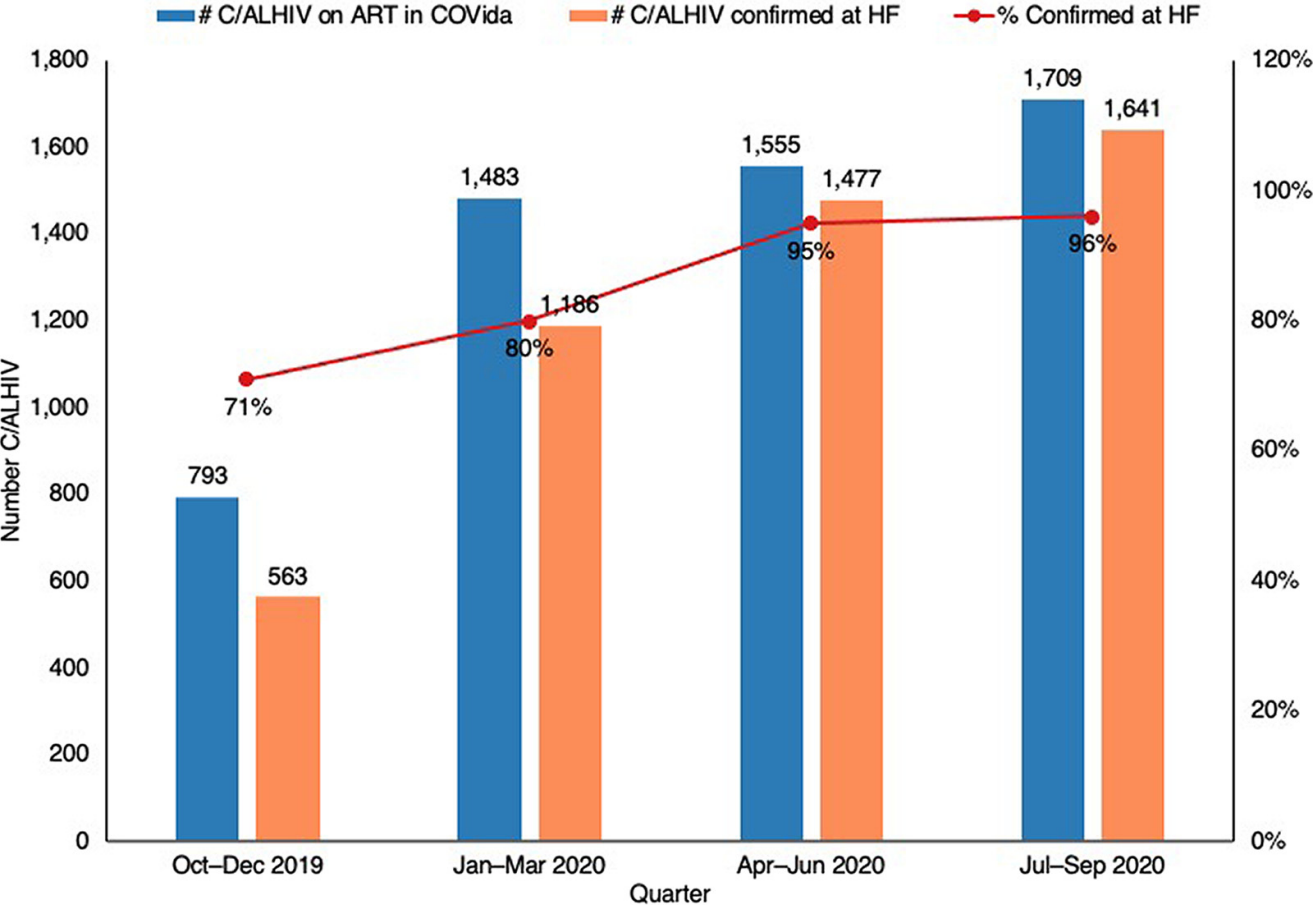
Comparison of Data From COVida Project vs. Health Facility on Children and Adolescents Living With HIV on Antiretroviral Therapy Abbreviations: ART, antiretroviral therapy; C/ALHIV, children and adolescents living with HIV; HF, health facility.

As of October 2019, there were substantial discrepancies identified in the numbers of C/ALHIV on ART as recorded in COVida project data and in clinical data for all but 1 of the 5 districts being assessed. However, the percent agreement on the number of C/ALHIV on treatment as recorded by the COVida project and health facilities in the districts improved considerably over the course of the pilot intervention (Figure 1). When the D2C intervention began, only 71% of the C/ALHIV on ART, as recorded by the health facility, were also documented on ART in COVida records. This percentage increased to 96% by the fourth quarter of the fiscal year (October to September).

The percent agreement on the number of C/ALHIV on treatment as recorded by the COVida project and health facilities in the districts improved considerably over the course of the pilot intervention.

### Viral Load Testing

In March 2020, VL data reviews were added to the D2C intervention and revealed gaps in VL testing. Among the 1,473 C/ALHIV confirmed to be on ART in health facilities, only 767 (52%) had a recorded VL test result in their medical records.

**FIGURE 2:**
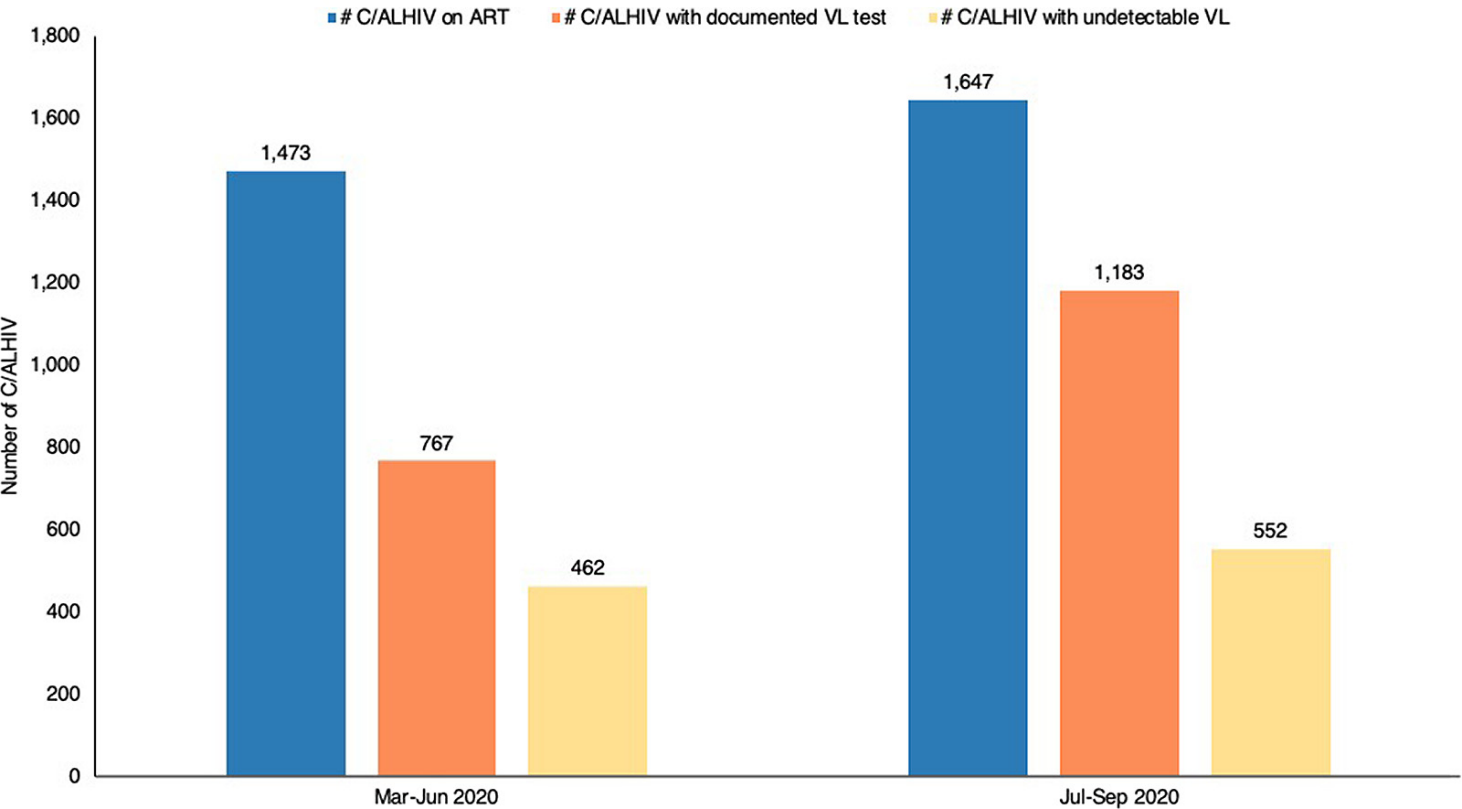
Viral Load Testing Results and Viral Suppression Among Children and Adolescents Living With HIV Enrolled in Services Within the 5 Districts Abbreviations: ART, antiretroviral therapy; C/ALHIV, children and adolescents living with HIV; VL, viral load.

The absolute numbers of those on ART and those with a recorded VL test increased between the March–June 2020 quarter and the July–September quarter. More importantly, the proportion of C/ALHIV on ART with a documented VL test increased to 72% (1,183 of 1,647) during this same period (Figure 2). The proportion of those with VL test results that had undetectable VLs decreased between the 2 time periods, from 60% (462 of 767) to 47% (552 of 1,183).

## LESSONS LEARNED

The D2C intervention provided a greater opportunity for community-based services to coordinate and collaborate with the clinic-based services serving the same population. By implementing this intervention, a number of challenges were identified for which correct actions were devised and implemented in collaboration between the 2 projects (Table 1).

The D2C intervention provided a greater opportunity for community-based services to coordinate and collaborate with the clinic-based services serving the same population.

**TABLE 1.** Challenges Identified Through the Data to Care Intervention and Corrective Actions Taken by the COVida Project

Challenge	Corrective/Improvement Actions Taken
Some C/ALHIV who were enrolled in the COVida project were not registered as ART patients in any of the health facilities in the 5 districts.	Case workers and their supervisors reviewed names in case management records, cross-checking with names on patient treatment cards, and then communicated findings to the health facilities.
Some HIV-positive children were registered as adults in the health facilities’ database, indicating discrepancies in children’s ages in the COVida project and the health facility databases.	Case workers and their supervisors cross-checked children’s age with their birth certificates and shared the correct information with the ESCALA data entry specialist based in the health facility. COVida project’s case management records were also corrected as needed.
Some C/ALHIV enrolled in the COVida project reported that they were taking their medications as prescribed yet were registered as being lost to follow-up in the health facilities.	Community case management workers were provided with lists of C/ALHIV designated as lost to follow-up by the health facilities and were able to find the C/ALHIV and bring them back to the health facilities to restart ART.
Some C/ALHIV registered by the COVida project as not being on ART were actually on ART and registered in a different health facility (to which they had transferred themselves).	COVida project updated its case management records to register the name of the new health facility.
Only about half of C/ALHIV had VL data.	The COVida project invited ESCALA staff to train the community case management workers to educate caregivers on VL testing and to encourage them to request this service in the health facility. The ESCALA project facilitated access to VL data in health facilities for COVida case managers to track children’s VL.
Only about half of those with VL results were virally suppressed.	The ESCALA project, with the COVida project, trained community case workers on ART adherence to improve monitoring of ART adherence and adherence counseling. Messaging on ART adherence for children and their caregivers was reinforced during home visits.

Abbreviations: ART, antiretroviral therapy; C/ALHIV, children and adolescents living with HIV; VL, viral load.

Anecdotally, COVida project managers also reported that the collaborative nature of the D2C intervention strengthened the relationship between those providing clinical care and those providing community-based ancillary care for C/ALHIV in Mozambique. They believed this helped contribute to improved outcomes for C/ALHIV.

Supporting C/ALHIV in their HIV treatment required that COVida case managers receive additional training on clinical aspects of HIV care to be able to counsel C/ALHIV and their caregivers. The COVida project trained the case managers to educate C/ALHIV and their families on ART adherence and VL testing to improve adherence support and to promote VL testing through community-based services.

### Going to Scale

Based on pilot intervention results, the D2C strategy was scaled up to an additional 104 health facilities across 5 additional districts in late 2020 and to all remaining COVida project areas in 2021. Although much of the work went smoothly, additional challenges arose as the intervention was implemented across a broader range of facilities (Table 2).

**TABLE 2.** Summary of Strategies to Address Challenges Identified During Scale-Up

**Challenges**	**Solutions**
Some C/ALHIV had incomplete or outdated information in the electronic patient tracking system	Health facilities allowed COVida staff to use patients’ physical files/records to complete pending information. Timeline for D2C activities was adjusted to happen after clinical partner’s data cleaning.
Case managers’ supervisors lacked time to conduct the D2C intervention in high volume sites	The COVida project allocated a full-time pediatric HIV supervisor in each district, whose salary was paid by the project, to facilitate and support the intervention and the updating of C/ALHIV data, monitor the progress of C/ALHIV, and provide specialized support for the most challenging cases.
Some clinical partners and health facilities’ staff expressed resistance to collaborate in implementing the D2C intervention	After the successful pilot experience in Inhambane, the COVida project presented the D2C standard operating procedures and tool to the USAID/Mozambique mission. To support the scale-up of this approach, the USAID/Mozambique Mission organized a meeting with clinical and OVC partners to promote the approach and requested that all clinical partners collaborate with OVC partners to scale it up.

Abbreviations: C/ALHIV, children and adolescents living with HIV; D2C, Data to Care; OVC, orphans and vulnerable children; USAID, U.S. Agency for International Development.

During the implementation of this D2C pilot intervention, the project saw an increase in the numbers of C/ALHIV correctly identified and enrolled in HIV care and treatment services, as well as a substantial increase in the proportion of C/ALHIV with known VL test results, necessary for appropriate treatment management. These improvements are critical to providing high-quality care and to the health and well-being of C/ALHIV. Of note, although the proportion of C/ALHIV on treatment who had a known VL test result increased over time, the proportion with a suppressed VL decreased. These results reinforced the critical role of VL testing in identifying C/ALHIV in need of adherence support to prevent treatment failure.

### Recommendations

The D2C intervention proved to be a beneficial approach for both the community-based project and health facilities because it helped both identify and correct data discrepancies and jointly address gaps related to both services. The intervention also increased awareness about the importance of collaborating and exchanging data between clinical partners and OVC programs. OVC programs can capitalize on the increased collaboration with health care services to reach more C/ALHIV with OVC programs.

The D2C intervention helped both the project and health facilities identify and correct data discrepancies and jointly address gaps related to both services.

Based on the experiences of the D2C approach implemented in Mozambique, HIV care and treatment programs in low- and middle-income countries should consider whether D2C may be a viable option for their own programs, particularly when multiple sources of data on the same PLHIV are available for triangulation. Support for this strategy in different settings and different service delivery configurations would be bolstered through additional research and/or evaluation efforts that more closely examine the effects of D2C on clinical outcomes among PLHIV in low- and middle-income countries.

## CONCLUSIONS

Data quality issues continue to plague many HIV care and treatment programs globally.^22^^,^^29^^–^^37^ Poor data quality can have direct detrimental effects on the efficient and equitable distribution of limited resources and for effective program management. However, more immediately, missing or incorrect data can undermine the health and well-being of PLHIV served by these programs. Identifying and adopting evidence-based intervention strategies in new settings are important to help address this important challenge. Real-time learning and implementation of the D2C approach within the context of a large HIV intervention program provided a useful opportunity to evaluate, refine, and scale up this evidence-based approach for improved patient outcomes.

## Supplementary Material

GHSP-D-23-00130-supplement.pdf

## Notes

**Peer Reviewed**

**First published online:** March 6, 2024.
