# Supplementary figures and images for: Adopting Data to Care to Identify and Address Gaps in Services for Children and Adolescents Living With HIV in Mozambique
Source: Glob Health Sci Pract. 2024 Apr 29;12(2):e2300130. doi: 10.9745/GHSP-D-23-00130 (PMC11057801; doi:10.9745/GHSP-D-23-00130)

## SUPPLEMENTARY MATERIAL

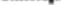[illegible]

Supplement: GHSP-D-23-00130-supplement.pdf [file GHSP-D-23-00130-supplement.pdf]
